# Supplementary material for: Predicting visual function by interpreting a neuronal wiring diagram
Source: Nature. 2024 Oct 2;634(8032):113–23. doi: 10.1038/s41586-024-07953-5 (PMC11446822; doi:10.1038/s41586-024-07953-5)
Supplement: Supplementary file 6 — CRF and ERF predictions for individual TmY4 and TmY9 cells. Analogous to Supplementary Data 3, but for TmY target types. Shown are the top four monosynaptic pathways, the strongest pathway passing through each of the top ten intermediary types (ranking from Extended Data Fig. 7), and the trisynaptic pathway Tm1–TmY–Dm3–TmY (see the section entitled Prediction of spatial normalization). [file 41586_2024_7953_MOESM6_ESM.zip › DataS4/TmY9q/720575940629801975.pdf]

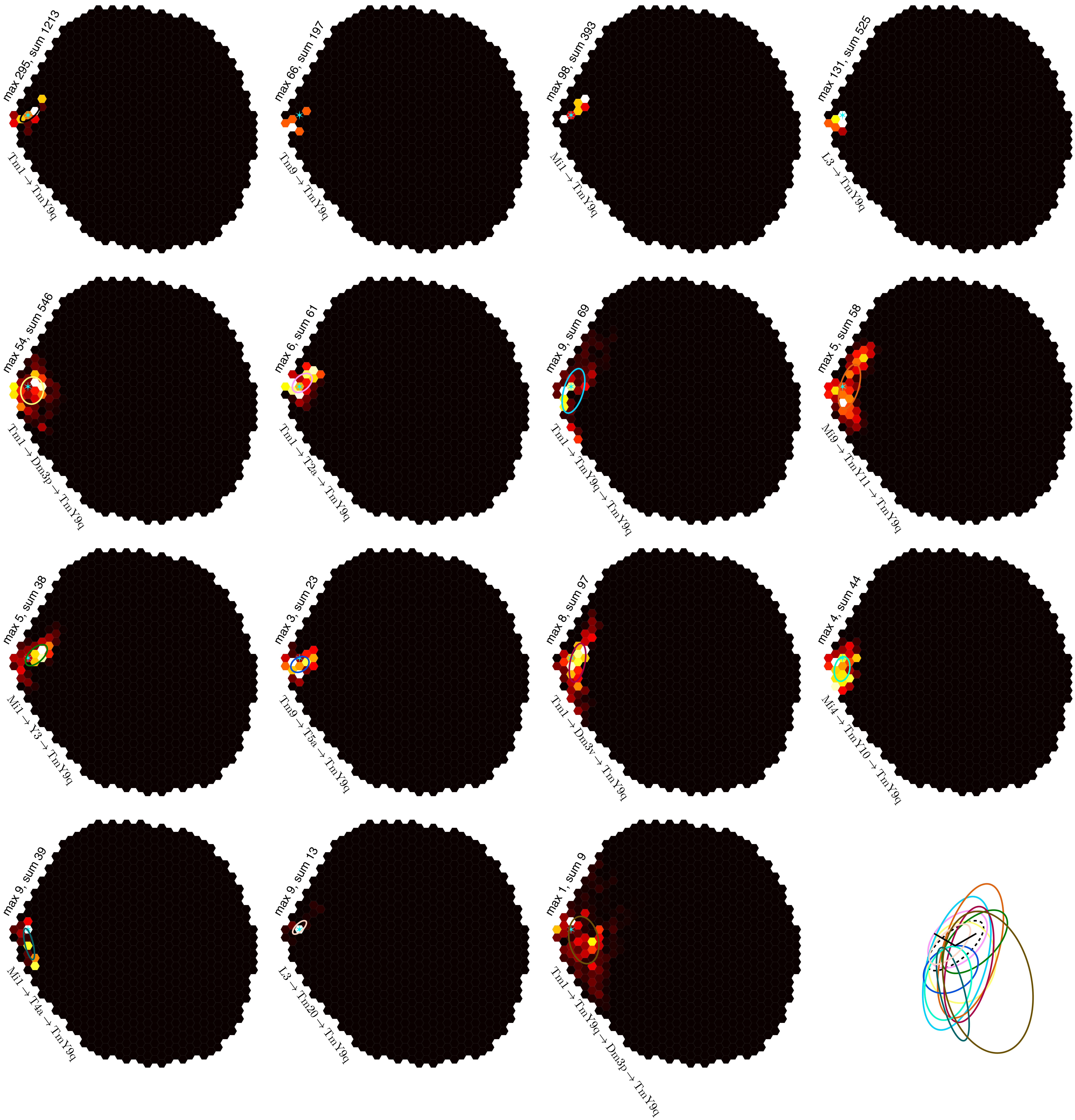

max 295, sum 1213
Tm1 → TmY9q
max 66, sum 197
Tm9 → TmY9q
max 98, sum 393
Mi1 → TmY9q
max 131, sum 525
L3 → TmY9q
max 54, sum 546
Tm1 → Dm3p → TmY9q
max 6, sum 61
Tm1 → T2a → TmY9q
max 9, sum 69
Tm1 → TmY9q → TmY9q
max 5, sum 58
Mi9 → TmY11 → TmY9q
max 5, sum 38
Mi1 → Y3 → TmY9q
max 3, sum 23
Tm9 → T5a → TmY9q
max 8, sum 97
Tm1 → Dm3v → TmY9q
max 4, sum 44
Mi4 → TmY10 → TmY9q
max 9, sum 39
Mi1 → T4a → TmY9q
max 9, sum 13
L3 → Tm20 → TmY9q
max 1, sum 9
Tm1 → TmY9q → Dm3p → TmY9q
